# Applications of lung cancer organoids in precision medicine: from bench to bedside

**DOI:** 10.1186/s12964-023-01332-9

**Published:** 2023-12-06

**Authors:** Huihui Li, Zexin Chen, Ning Chen, Yun Fan, Yaping Xu, Xiaoling Xu

**Affiliations:** 1 Department of Medical Thoracic Oncology, Zhejiang Cancer Hospital, Hangzhou Institute of Medicine (HIM), Chinese Academy of Sciences, Hangzhou, 310022 Zhejiang China; 2 Postgraduate Training Base Alliance, Wenzhou Medical University, Wenzhou, 325035 Zhejiang China; 3 Guangdong Research Center of Organoid Engineering and Technology, Guangzhou, 510535 Guangdong China; 4 Department of Oncology, The Second Clinical Medical College, Zhejiang Chinese Medical University, Hangzhou, 310053 China; 5 Department of Radiation Oncology, Shanghai Pulmonary Hospital, Tongji University School of Medicine, Shanghai, 200433 China

## Abstract

As the leading cause of cancer-related mortality, lung cancer continues to pose a menacing threat to human health worldwide. Lung cancer treatment options primarily rely on chemoradiotherapy, surgery, targeted therapy, or immunotherapy. Despite significant progress in research and treatment, the 5-year survival rate for lung cancer patients is only 10–20%. There is an urgent need to develop more reliable preclinical models and valid therapeutic approaches. Patient-derived organoids with highly reduced tumour heterogeneity have emerged as a promising model for high-throughput drug screening to guide treatment of lung cancer patients. Organoid technology offers a novel platform for disease modelling, biobanking and drug development. The expected benefit of organoids is for cancer patients as the subsequent precision medicine technology. Over the past few years, numerous basic and clinical studies have been conducted on lung cancer organoids, highlighting the significant contributions of this technique. This review comprehensively examines the current state-of-the-art technologies and applications relevant to the formation of lung cancer organoids, as well as the potential of organoids in precision medicine and drug testing.

Video Abstract

**Supplementary Information:**

The online version contains supplementary material available at 10.1186/s12964-023-01332-9.

## Background

Lung cancer is currently the most common type of cancer worldwide, with the highest mortality rate compared to other forms of cancer. It accounts for approximately 11.4% of all cancer cases and 18.0% of cancer-related deaths worldwide [1]. To provide sufficient decision-making evidence and judge prognosis, emerging methods such as genomics and microbiomics should be actively applied in addition to traditional diagnostic methods. The core of precision medicine involves diagnosis through underlying molecular analysis methods such as genetic testing. This allows for development of novel therapeutic avenues that differ from surgery and chemotherapy, including targeted therapy based on specific tumor driver genes and immunotherapy based on tumour mutational burden (TMB) [2].

In recent years, notable progress has been made in the field of targeted anticancer drugs and immunotherapy for lung cancer, but challenges regarding drug efficacy, toxicity and drug resistance remain. For example, lung adenocarcinoma exhibits marked inter- and intratumoral heterogeneity, which may lead to treatment failure and resistance development [3]. Therefore, it is crucial to design in vivo and in vitro model systems that can precisely imitate tumours to effectively analyse tumour in vitro culture, cell types, and drug sensitivity. The use of two-dimensional (2D) cell culture is widespread in lung cancer research for drug screening purposes, however, it does not encompass the in vivo tumor microenvironment (TME) [4]. A accumulation of genetic and epigenetic aberrations [5] during the in vitro process may negatively impact the culture of stem cells and diversity of cell types [6]. While human cancer cells that have been cultured in 2D and immortalized lose their phenotypic and genetic variability, tumour xenografts derived from patients (PDX) can largely preserve the original tumour's heterogeneity [7]. It presents a potential platform for testing the effectiveness of personalized anticancer drugs in drug screening and the development of new drugs. However, this approach is not suitable for large-scale drug discovery screening due to its high cost and time consumption. As a result, endeavors have been made to produce patient-derived organoids (PDOs), a three-dimensional (3D) in vitro model. The model manifests molecular and morphological characteristics that are more akin to those in vivo when compared to 2D cell culture. Over the past decade, numerous studies have initiated assessment of anticancer medication using PDOs as a viable approach to determine optimal drugs for patients who satisfy multiple treatment requirements. This review focuses on the comparison of PDO models with traditional cell lines and PDX models, with a detailed explanation of the usage, constraints, and future outlook of lung cancer PDOs.

## Overview of lung cancer organoid models

**Fig. 1:**
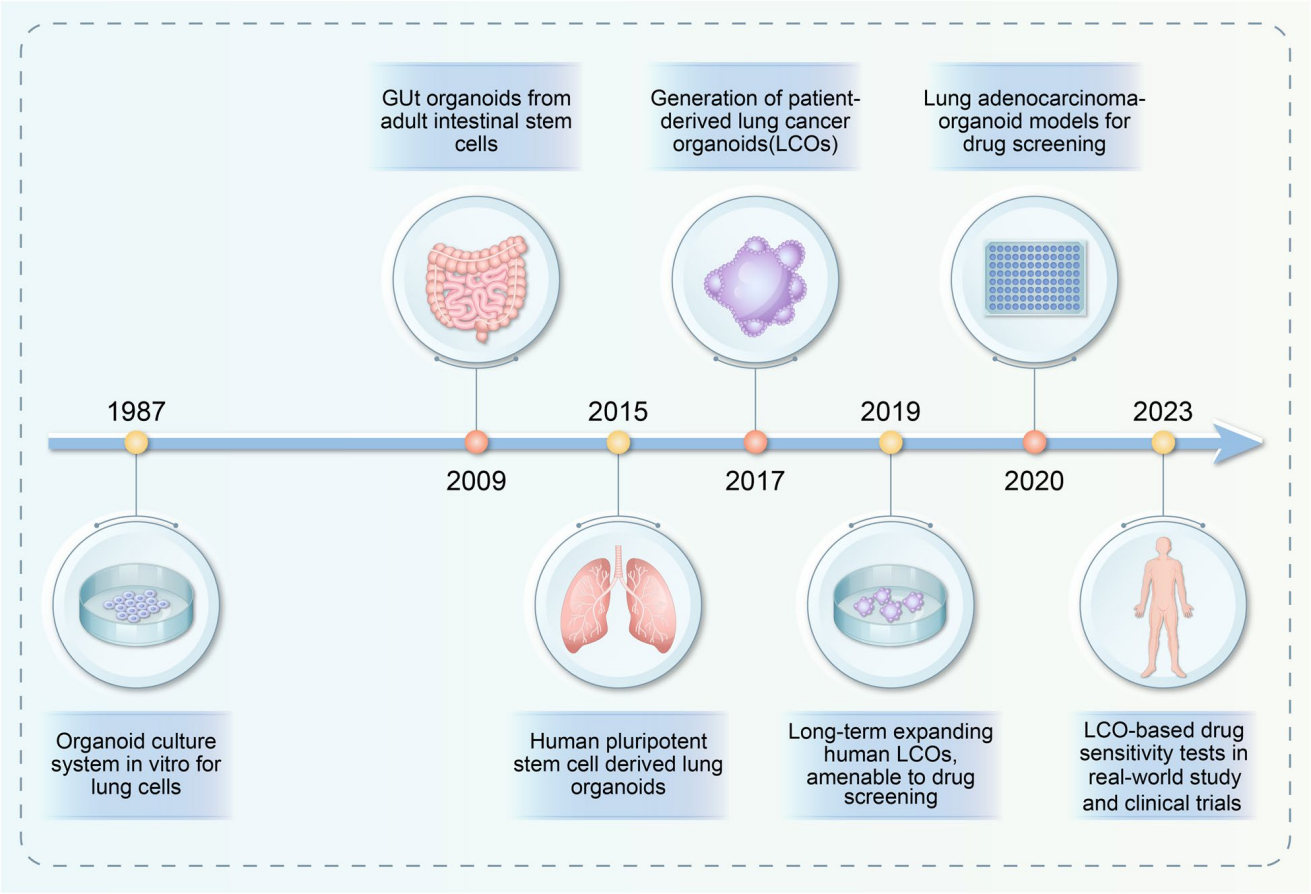
Timeline of the lung cancer organoid culture system related to drug screening

Organoids are in vitro 3D mini "organs" that highly mimic the pathophysiological system of the human body and can be created from embryonic stem cells (ESCs) [8], spermatogonial stem cells (SSCs) [9], and pluripotent stem cells (iPSCs) [10]. Through a self-assembly process, these organoids form with the aid of the stemness of cells sourced from a patient. While the in vitro culture of animal cells and organs has been a topic of scientific concern for almost a century, the first in vitro organoid culture of lungs was reported in 1987 [11]. In the past decade, there has been considerable advancement in organoid technology (Fig. 1). Organoid technology has gained attention since 2009, when the Hans Clever laboratory successfully self-organized intestinal organoids with intestinal crypt-villus structures in vitro. This was achieved by using individual murine LGR5 + intestinal stem cells [12]. Lung organoids induced by pluripotent stem cells (hPSCs) were successfully cultured in 2015 [13]. In 2017, Pauli et al. created 56 patient-derived organoid cultures for 769 patients, which were used for high-throughput drug screening. This event marked the first appearance of lung cancer organoids (LCOs) in the literature and laid the groundwork for personalized treatment of lung cancer [14]. In 2019, LCO lines generated by Sachs retained histopathological and mutational characteristics of the original tumour and were suitable for drug screening experiments [15].

To date, biobanks for lung cancer organoids have been established from multiple subtypes of lung cancer tissues of primary biopsy or surgery, including adenocarcinoma [16], squamous cell carcinoma [17] and small cell carcinoma [18].For lung adenocarcinoma (LUAD), Li et al. successfully developed 12 LUAD organoid lines using a 3D culture medium. The majority of the organoids exhibited positive staining for adenocarcinoma markers such as TTF-1 and Napsin A, thus demonstrating the histopathological characteristics consistent with the parental cancers [16]. It was found that multiplex gene editing of mouse lung organoids using the CRISPR-Cas9 system can effectively and rapidly generate lung squamous cell carcinoma (LSCC) that closely mimic the human disease at the genomic and phenotypic level [17]. Jeong et al. generated patient-derived tumor organoids from small cell lung cancer (SCLC) and subjected them to long-term expansion with the addition of WNT3A or R-spondin1. Through comprehensive genetic and histopathological analyses, it was observed that organoids maintained the genetic profiles, molecular characteristics, and morphological architectures of the original tumors [18]. Further prospective studies are required to assess the efficacy of clinical treatment decisions, despite promising results of translational research on lung cancer organoid models and precision medicine.

## Comparison of organoids with traditional cell models and PDX models (Table 1)

**Table 1:**
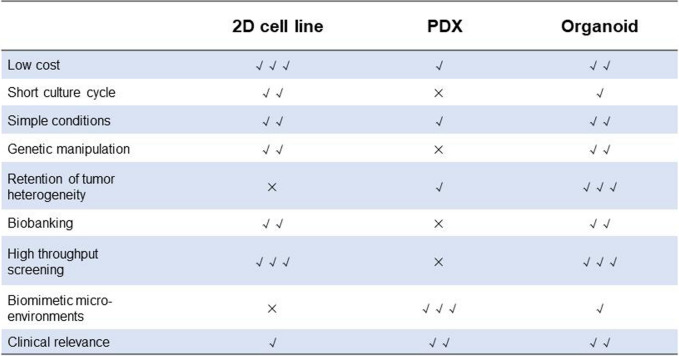
Comparison of advantages and disadvantages of 2D cell line, Patient-derived xenograft (PDX) model and organoids

### Cell line

Representative of traditional two-dimensional culture, cell lines remain the most common disease models. While cell lines have the benefits of low cost, high throughput, and simple culture conditions, their disadvantages have become increasingly apparent. One important property of cell lines is the unavoidable genetic drift that occurs over time, resulting in progressive accumulation of genetic mutations. This will cause significant damage to further studies if the phenotypic and genetic heterogeneity in tumour cells is altered [19]. Additionally, cell lines grown in culture dishes represent an oversimplified and detached biological system that may not accurately represent the complexity of the in vivo environment. Cell lines lack complex components and interactions involving the TME, such as stromal cells, immune cells, and endothelial cells, which are critical for controlling tumour growth, metastasis, and angiogenesis [20]. Furthermore, cell lines in flat culture cannot detect tumour-matrix interactions between cells and the extracellular matrix (ECM), severely limiting their ability to mimic the in vivo environment.

### Patient-derived xenografts (PDX)

PDX models are frequently generated using immunodeficient or humanized mice, which are transplanted in situ or ectopically once the tumour load reaches a certain level. Compared with cell lines, PDX models better preserve key features and interactions between the tumour and its stroma [21]. In addition, PDX models offer a considerable number of cancer types to study [22], with many tumours having been stably modelled, including lung cancer [23]. However, genomic variants especially in cancer-related genes appear to be higher in PDX. Due to the risk of tumour rejection, PDX models are often constructed using immunodeficient NOD/SCID or NOD/NSG mice, which makes them unsuitable for evaluating the efficacy of immunotherapy [24]. Furthermore, PDX models have a long culture cycle [25] and are expensive [26], making it difficult to adapt to high-throughput drug screening platforms. Coupled with the limitations of gene editing technology [27] and the high rate of transplantation failure, the application and promotion of PDX models are hampered. Even if some researchers hold promise for PDX models constructed with juvenile or adult zebrafish as subjects, it is challenging to carry out [28] because of their low clinical use rate and nonmammalian reasons.

### Organoids

The ability of organoids to self-renew and self-proliferate while retaining the specific functions and structures of native tissues has generated significant interest in their use for disease modelling [29]. The specific advantages are as follows. ① The system is cost-effective and easy to build [30]. Compared with PDXs, both the initial construction and subsequent maintenance of organoids are cheaper. Additionally, they are more comprehensive than cell lines in constructing disease models, thus making it possible to establish a living biobank. ② Gene editing technologies, such as CRISPR/Cas9, can be utilized to genetically modify organoids for experimental purposes. This includes inducing autologous tumours to rapidly reproduce gene profiles, performing gene screening, and applying it to antibody development [31–33]. ③ Organoids reserve their morphology and genetic characteristics after long-term culture and expansion [34]. Moreover, the low construction cost of organoids provides favourable conditions for high-throughput studies [35]. ④ The matrix structure and function of this model can affect tumour tissue and structure, treatment response, multicellular resistance (MCR) and drug penetration. However, each biological model has its limitations. For instance, organoids cannot fully replicate human organs and may have some defects, such as a gradual decrease with an increase in passage number and a lack of matrix and vascular system.

## Culture and application techniques of organoids

### Culture of organoids

**Fig. 2:**
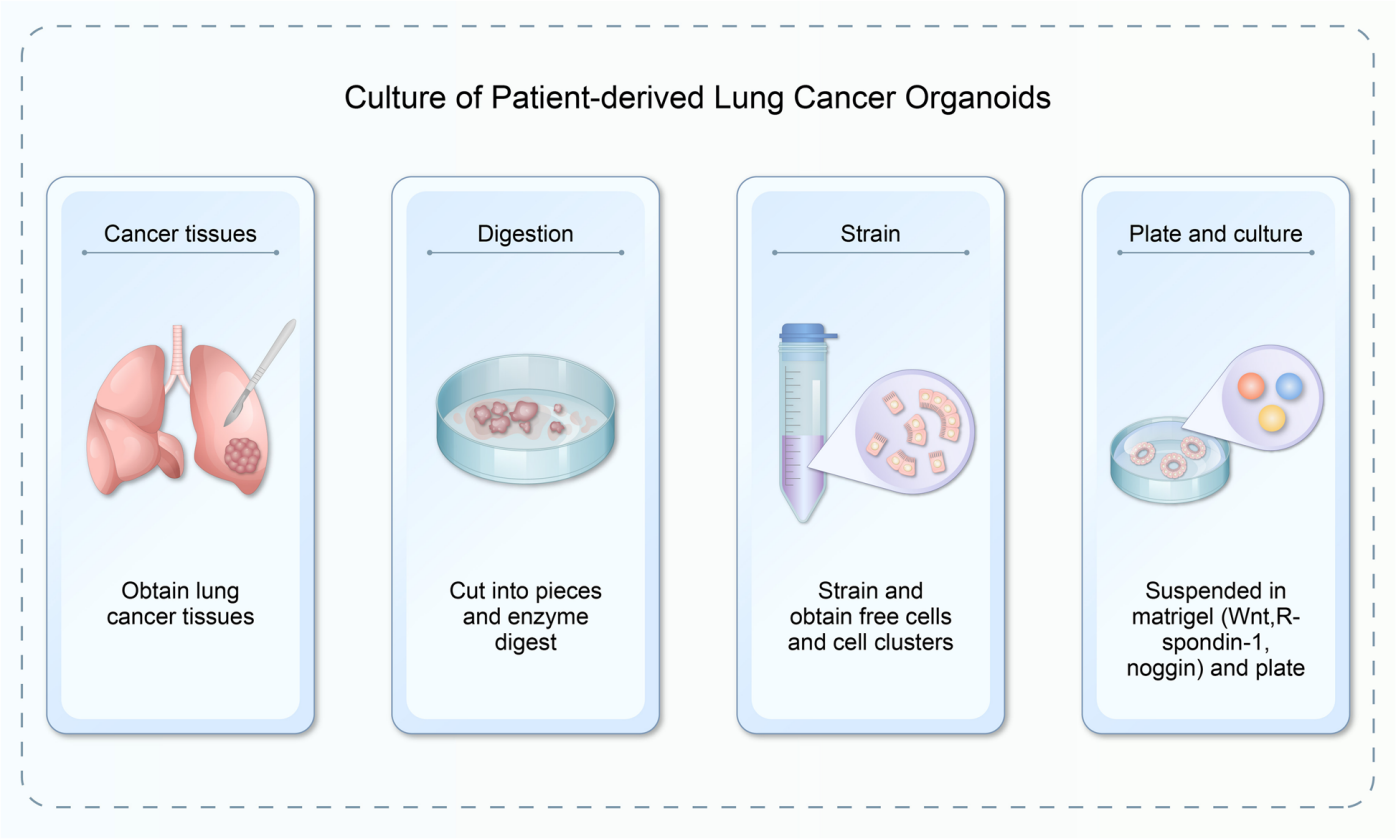
Establishment of the lung cancer organoid model

The core principle that governs development of diverse normal tissues and tumour organoids remains largely uniform, primarily hinging on creation of a three-dimensional microenvironment through matrix adhesives. Various nutrients that maintain cell stemness are added to the culture system to promote cell growth and development. Following dissociation of cancer tissues, several procedures, including physical cutting, chemical digestion, and filtration, are necessary to degrade a suitable lung cancer sample into unbound cells and cell clusters. Then they are suspended in a liquid extracellular matrix (ECM). The mixed matrix gel is inoculated on plates and allowed to solidify (Fig. 2). Afterwards, a specific medium is added and lung cancer organoids can be obtained after 1–2 weeks of culture.

Establishment of organoids has the following priorities: ① find and obtain tissue/organ stem cells; ② create cultivation environment; and ③ maintain cell stemness. Most of the cells used for organoid culture are derived from stem cells including tumour stem cells derived from the desired tissue or organ because of their strong cell stemness. For example, the addition of low concentrations of PF-127 to the cell culture medium has been found to promote the differentiation of human embryonic stem cells (hESCs) into organoids [36]. In 2021, the researchers induced in vitro human induced pluripotent stem cells (hiPSCs) to develop a brain organ model and established an in vitro model of sporadic Alzheimer's disease [37]. Matrigel is composed of laminin, nidogen and number growth factors. However, the contents of Wnt, R-spondin-1 and Noggin alone are not sufficient to support organoid culture, and additional factors such as EGF and FGF are usually necessary [38–40]. The Wnt/β-catenin pathway is a highly conserved signalling pathway and plays a crucial role in embryonic development, cell proliferation, differentiation, neurodevelopment, and carcinogenesis. This highlights the indispensability of Wnt in maintaining cell stemness [41]. R-spondin-1 activates the Wnt/β-catenin signalling pathway by increasing its own expression. It also functions synergistically with Wnt, Fzd, and LRP6 to regulate Wnt [38]. Noggin, an extremely important regulator of the Wnt signalling pathway, is indispensable for almost all organoids [42].

### Frontiers of organoid culture in lung cancer (Table 2)

**Table 2:**
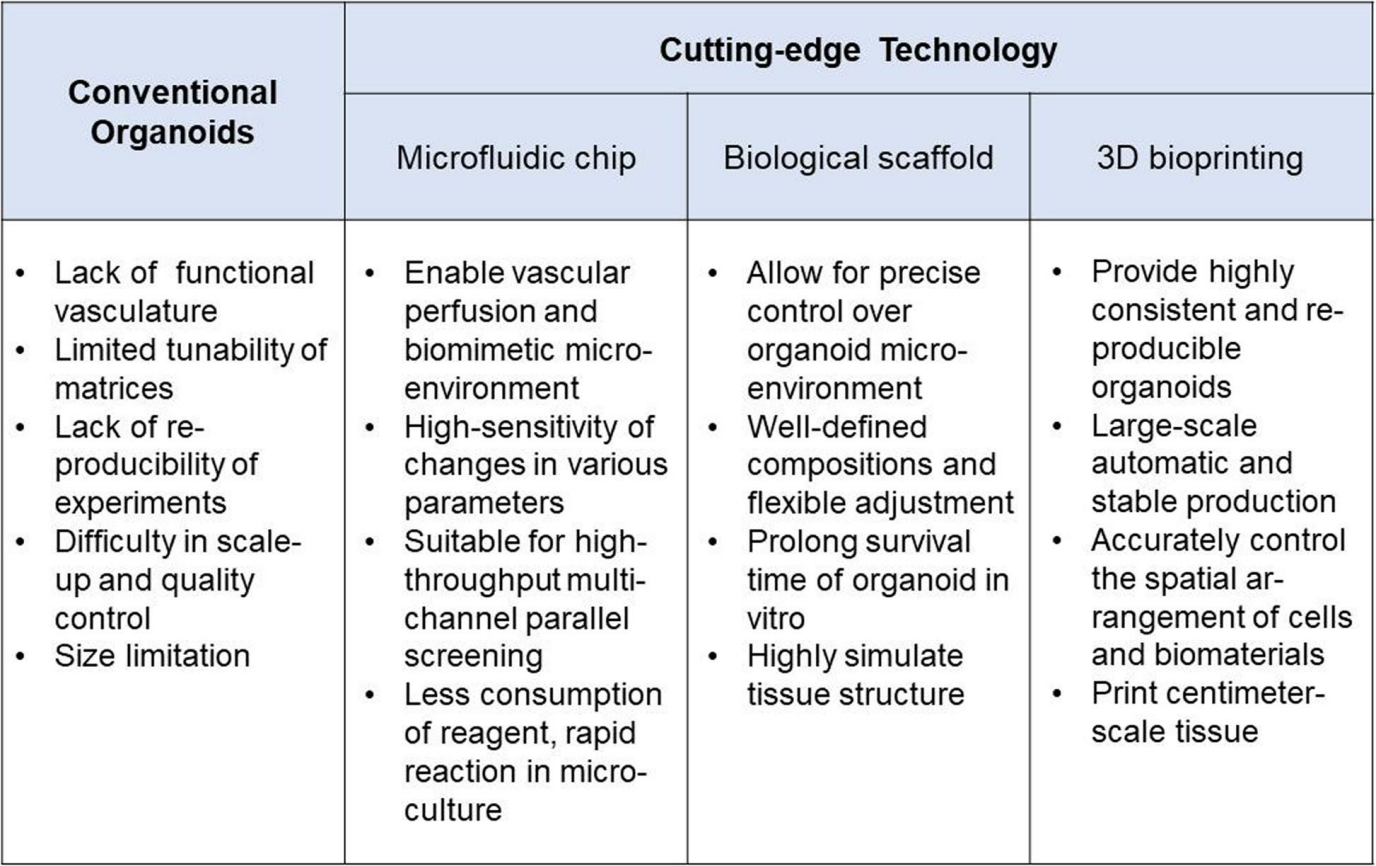
Comparison among the conventional organoids to the currently applied technologies

#### Microfluidic chip

Traditional organoid culture systems have limitations in terms of variability and scalability. Additionally, existing technologies struggle to achieve large-scale production of homogeneous organoids for drug screening. To improve the accuracy and stability of organoid research, researchers have been studying microfluidic devices with microfluidic chips as the core. These chips are used to construct and simulate the human tissue microenvironment, creating a microphysiological system that closely resembles the human body. Through dynamic flow in microfluidic devices for waste exchange, oxygen and nutrient supply, organoids can be precisely managed and uniformly cultured in large numbers while preserving a complex microenvironment [43]. The device is designed to mimic the structure of living tissue in a laboratory setting, with a continuous perfusion chamber and multiple layers of cells. This technology, known as organoid microarrays, has gained attention from the medical community in recent years due to its ability to model diseases such as for cancer modelling [44], drug screening, and preclinical research. Liu and colleagues combined microfluidic chips with two cutting-edge technologies for tumour organoids [45] and performed drug sensitivity testing with 100% accuracy and specificity. Hu et al. used the characteristics and advantages of microfluidic chips to reduce the time needed for construction and application of tumour organoids, with great prospects for clinical translation [45]. One study showed that patient-derived mesothelioma organoids and microfluidic devices can be combined to screen chemotherapy and precisely detect targeted agents through genetic analysis [46]. In addition, Jung et al. proposed a microfluidic chip that can culture lung cancer organoids and test drug susceptibility directly on the microphysiological system (MPS) [47]. These strategies, when combined with microfluidic chips, may enable screening of more complex, scalable, and precise anticancer drugs in cancer drug research.

#### Biological scaffold

To culture organoids, hydrogels must provide the necessary physical factors such as space and pressure stimulation, as well as the nutrients required for organoid growth. This creates an environment that mimics the natural physiological conditions in which stem cells are found in vivo, and provides cancer cells with an extracellular component that is rich in nutrients and can stably support 3D structures. The hydrogel acts as a scaffold for stem cells to adhere to and self-assemble into specific organoids. Because this hydrogel is man-made, it is easy to control its chemical composition and critical properties and ensure consistency without posing a risk of infection or immune response; it is therefore significantly better than traditional Matrigel. Wilkinson et al. developed a self-assembling lung organoid platform [48] with various types of pneumocytes by applying alginate saline gel beads with collagen function and used this platform to establish an idiopathic pulmonary fibrosis (IPF) model. Based on the bioreactor-harvested A549 lung adenocarcinoma cell line rat-derived cell-free lung was proposed as a scaffold for a 3D lung cancer model [49]. A 3D model of the effect of lung cancer on muscle atrophy was presented by Mondrinos; the study also established a chemotherapy-resistant lung cancer model using the cisplatin-resistant A549 cell line established on 3D type I collagen hydrogel [50].

#### 3D bioprinting

3D bioprinting is a tissue engineering method that uses software to rapidly manufacture biomedical products by layering and controlling the positioning and assembly of biomaterials. This technology has the potential to replace artificial methods and construct complex heterotypic tissues, improve automation and biomimicry in model quality, and provide highly consistent, reproducible, and scalable organoids. As a result, bioprinting technology is showing great potential in high-throughput screening and individualized precision treatment. In their study, Melissa and colleagues were able to culture renal organoids using 3D bioprinting technology achieving morphology, component cell types, and gene expression levels comparable to those constructed manually [51]. This technology has the potential to produce organoids with high-throughput and controllable precision. In 2022, the Clark team developed an optimized organoid immersion bioprinting method and applied it to drug screening for brain metastases from lung adenocarcinoma. The combination of the two technologies can fully embody the absolute advantages of organoids in high-throughput research and realize large-scale automatic and stable production of organoid biological models. Despite some progress in 3D bioprinting, many challenges remain when developing controllable and sufficiently complex organoids, such as bioink materials lacking suitable printed cells [52]. For this reason, more studies are needed to attain practical application of bioprinted organoids.

## Application of lung and lung cancer organoids

### Personalized medicine for lung cancer

Organoid technology has been acknowledged as a promising biotechnology in precision medicine. Vlachogiannis and colleagues conducted a pioneering study on organoid drug sensitivity testing and in vivo consistency in gastrointestinal tumours, yielding remarkable results [53]. Subsequent studies on other cancers including breast [54] and pancreatic [55] cancers have been carried out. Recently, Wang conducted a real-world study on organoid drug sensitivity and the consistency of clinical efficacy for advanced lung cancer. The study identified concordance rates that exceeded 80%. However, the accuracy of organoid prediction for different types of drugs was found to be variable [56]. In addition, there are some individual case reports on predicting the efficacy of LCOs on patients [57, 58]. Overall, the current research on LCOs is still in the observational study stage. The results will promote development of future clinical trials, particularly interventional clinical trials. They will also continue to explore the prospects and applications of organoids for individualized medicine.

### Drug discovery screening for lung caner

In the past, the development and screening of many antineoplastic agents was performed using standard cell lines, but they proved ineffective in clinical studies [59]. However, organoids are highly adapted to the normal human physiological status and can be used in high-throughput studies. FDA Modernization Act 2.0, which was enacted in December 2022, legally allows drug developers to utilize new models as a substitute for animal experiments. This development will aid in rapid advancement of organoid technology in drug research and development, as well as in screening of new drugs [60]. The study conducted by Hu et al. showed that hundreds of LCOs were capable of producing clinically significant drug responses within a week, providing a technically feasible method for predicting patient-specific drug responses in the clinical setting [45]. A phase II clinical study showed that pyrotinib had better efficacy than afatinib in an organoid model for patients with HER-2 exon mutated lung cancer, indicating that LCOs can accurately predict treatment response [61]. The study of new antitumour drugs through organoids will also be an important development trend in the future. An increasing number of studies are using organoids to screen potential antitumour compounds, including small molecules, macromolecular polysaccharides and antibodies [62–65]. However, there are still few studies on drug screening through LCOs, especially drugs with rare mutations, which holds broad prospects and greatly reduces screening time.

### Establishment of biobanking

Biobanks have been established due to their advantages of low cost and easy maintenance organoids-based. However, effective diagnosis and treatment programs for glioblastoma (GBM) tumours are challenging due to their high inter- and intratumoural heterogeneity. To address this issue, Jacob established glioblastoma organoid (GBO) biobanks, which retain the key characteristics of GBM. This resourceful biobank provides a rich source for inositol research and translational research in GBM, further reflecting the significant advantages of GBO in testing personalized treatment [66]. The establishment of Liver Cancer Organoid Biobank in 2023 aimed to provide a comprehensive representation of the molecular and histological features of different types of liver cancers. This was achieved through multiomics profiling, which included epigenomic, genomic, proteomic, and transcriptomic analyses. This study presents a comprehensive pharmaco-proteogenomic analysis of liver cancer organoids, which may advance predictive modeling of biological mechanisms of drug responses and potential drug combinations [67]. In terms of lung cancer organoids, Kim and colleagues established an in vivo biobank consisting of 80 LCOs derived from five histological subtypes, including lung cancer and nonneoplastic bronchial mucosa. This study demonstrated that changes and responses of LCOs to drug sensitivity depend on genetic alterations. Drug trials against EGFR-mutated organoids and MET-amplified organoids have been conducted to predict the efficacy of patient-specific treatments [68].

### Disease model development

Organoid models have been established for various tumours and diseases, including PDOs applied to primary cultures of lung cancer cells. These models serve as ex vivo models to accurately reproduce clinical responses to chemotherapy and immunotherapy in precision treatment [69]. Zhang conducted a study in which patient-derived NSCLC organoids were established and confirmed that tumour cytological features were stable and that specific markers remained constant over serial passages [70]. Miura et al. successfully cultured lung organoids and induced overexpression of the oncoprotein human epidermal growth factor receptor 2 (HER2)/ERBB2 using doxycycline. This indicates that organoids can be applied to construct preclinical models in HER-2 overexpressing lung cancers [71]. Park et al. acquired human bronchial epithelial cells and performed gene manipulation, including upregulation of oncogenes in myelocytoma and entry of B-cell leukaemia/lymphoma 2. Inoculation of the resulting organoids into immunosuppressed mice produced tumours that closely resembled SCLC [72]. Multiple disease models have been constructed by using organoids of human distal lung tissue, such as interstitial lung disease, lung cancer, and respiratory syncytial virus infection models [15].

### Organoids in applications of lung cancer basic research (Fig. 3)

**Fig. 3:**
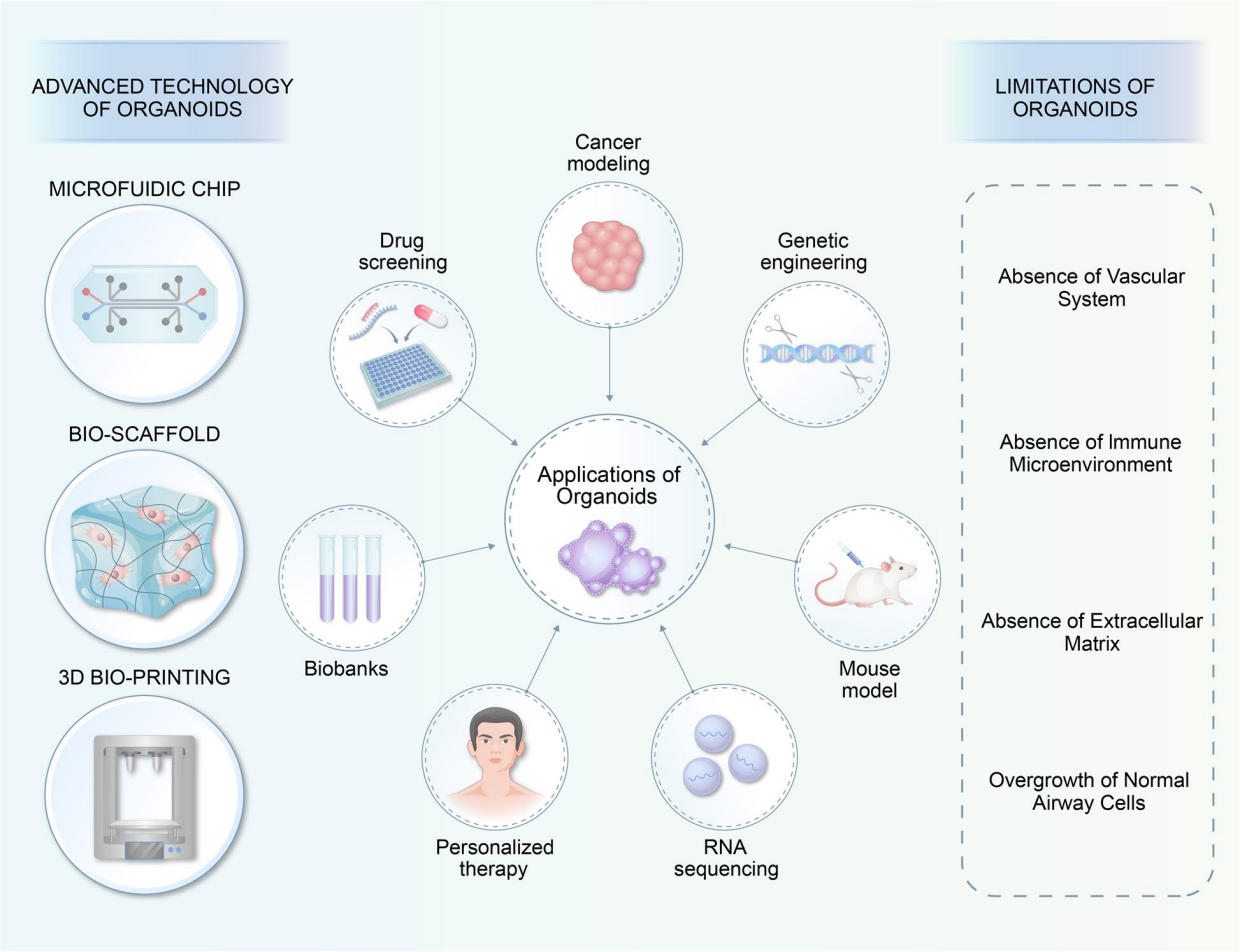
Applications, frontiers and challenges of organoids in basic and clinical research

#### Gene-editing techniques

CRISPR‒Cas9 is an adaptive immune defence that evolved over time in prokaryotes to combat invading viruses and exogenous DNA. CRISPR‒Cas9 technology enables specific DNA modification of targeted genes, making it a valuable tool in gene screening and disease treatment. Hai and colleagues used CRISPR‒Cas9 to knock out multiple oncogenes from Cre-dependent SOX2 knock-in mouse lung organs and created a lung squamous cell carcinoma model that resembles human disease at genomic and phenotypical levels [17]. PD-1 inhibitors have been found to enhance WEE1 inhibition and promote cytotoxic T-cell-mediated clearance of tumour cells, thereby reducing accumulation of tumour infiltrating neutrophils. It is suggested that organoids can be used in research on combination immunotherapy. In 2020, Dekkers knocked out breast cancer-associated oncogenes with the help of CRISPR/Cas9 and successfully evaluated the tumourigenic capacity of individual gene mutations, contributing to further identification of new cancer driver genes and new drug target discovery [32].

#### Single-cell sequencing

High-throughput single-cell sequencing technology has become a crucial tool in exploring biological questions such as tumour heterogeneity through analysis of the genetic material of individual cells. This method allows for identification of heterogeneous cells through cell fractionation and study of interactions between tumour cells [73]. For example, single-cell RNA sequencing of hepatobiliary tumour organoids revealed that drug resistance can be caused by interactions between different intratumour subpopulations [74]. An in vitro colorectal cancer organoid culture system was systematically evaluated at the single-cell level, showing that organoids completely recapitulate DNA methylation patterns and major gene expression profiles [75]. Furthermore, RNA sequencing (RNA-seq) results for NSCLC cells treated with fangchinoline (Fan) revealed that Fan effectively suppresses NADP + metabolic processes and inhibits the Akt-mTOR signalling pathway by directly suppressing Akt-mTOR-promoted NOX-4 degradation [76]. The combination of single-cell transcriptome sequencing with organoids enables analysing similarities between organoids and real organs at the cellular, genetic and molecular levels, monitor dynamic transcription and studying cell interactions and signalling communication.

#### Organoids and mouse animal model

Na et al. constructed a primary, in situ, driver gene defined mouse model of SCLC using organoid culture, gene editing and orthotopic transplantation. The model is expected to be applied to basic and translational studies of SCLC. In addition, the team found that loss of the epigenetic gene KMT2C promotes SCLC metastasis and malignant progression. It is anticipated that SAM can prolong survival periods and improve quality of life for patients with KMT2C mutations, providing a new therapeutic target for SCLC patients [77]. Lung squamous cell carcinoma organoids were established using genetically engineered mice, which revealed that WEE1 inhibition enhances the antitumour activity of anti-PD-1 monotherapy [17].

## Limitations and challenges of organoid techniques

Application of patient-derived organoids is now prevalent in basic and translational studies of lung cancer. However, certain restrictions currently limit the potential applications of organoids in personalized medicine and drug development. The absence of a vascular system, a distinct immune microenvironment, and variations in cell types and extracellular matrix from in vivo tissues and organs present obstacles for organoids with regard to replicating the authentic microenvironment and drug reaction.

### Absence of a vascular system

Currently, most organoids lack in vitro vasculature, leading to necrosis in exploding tumours. Nevertheless, the 3D bioprinting technique has effectively generated centimetre-scale intestine-like tissues that are highly biomimetic, comprising tubular structures, branched vessels and small intestinal epithelial-like crypts [78]. A study carried out by Claudia and colleagues focused on investigating the angiogenic ability of cancer cells in a specific environment through introduction of certain substances to hydrogels. It has been found that upregulation of IL-8 significantly contributes to angiogenesis in tumour tissue [79]. In addition, comparative analyses were carried out by the team between blood vessel organoids and tumour vascular organoids. Research on the distinct mechanisms of tumour angiogenesis has the potential to enhance the vascular system of tumour organoids in personalized medicine [80].

### Absence of the immune microenvironment

Constructing tumour organoids that accurately mimic interactions between tumour cells and extracellular matrix is a significant challenge due to the complex cellular components present in the TME. Macrophages, which are abundant in the tumour immune microenvironment, have been found to promote cancer cell invasion through the COX-2/PGE2/β-catenin signalling pathway by inducing the epithelial-mesenchymal transition (EMT). Coculture experiments with human lung cancer cells exhibit this phenomenon [81]. Consequently, studying of indirect interactions between solid tumour cells and infiltrating immune cells in the immune microenvironment has been enhanced through use of macrophage coculture. Various methods have been proposed to maintain the immune microenvironment, such as the air–liquid interface [82] and in vivo coculture of tumour organoids with T cells from peripheral blood lymphocytes [83].

### Absence of the extracellular matrix

The extracellular matrix (ECM) is composed of several proteins including collagen, laminin, and cell cohesion [84]. Due to its complex components, the ECM plays a crucial role in determining cell phenotypes in cancer tissues. Abnormal changes in ECM degradation and remodelling are commonly observed in tumour tissues, making it an topic of interest for constructing tumour organoids [85]. To maintain the organoid environment, researchers are enriching the composition of hydrogels with specific components in an effort to build the ECM in vitro [86]. Some organoid studies have developed decellularized tissues (acellular matrix) with little effect on the structure and composition of ECM [87], and they are currently being applied in cancer invasion and colonization studies [88]. To investigate breast cancer cell colonization in the lung, Xu and colleagues established an in vitro model of lung colonization using an acellular lung matrix. Combined with organoid models, the technique can well simulate cancer progression and predict treatment response [89].

### Overgrowth of normal airway cells

Dijkstra et al. combined genetic and histomorphometric analyses, and observed overgrowth of normal airway organoids in most lung cancer organoids derived from over 70 NSCLC samples [90]. The rate of establishment for lung cancer organoid samples was 41%, whereas it was only 17% for pure lung cancer, limiting its potential use in precision therapy. It was discovered through gene sequencing and bioinformatics technology that the efficacy of LCOs is contingent upon the ratio of neoplastic cells in the original tissue [91]. Sachs proposed using the MDM2 antagonist Nutlin-3a to select TP53-mutated LCOs and prevent overgrowth of normal airway organs. However, this approach is not applicable to LCOs without TP53 mutations or normal airway organoids with TP53 mutations. To prevent pollution by normal cells in the culture system, Hu's team utilized a medium that lacks essential nutrients for ordinary airway organoid development. Despite not achieving 100% purity, the team identified dominant cancer cell organoid growth [45]. Moreover, Wang conducted drug sensitivity testing on LCOs derived from pleural effusions of various patients to predict clinical response. This method has potential for enhancing the purity of LCOs [56]. Nevertheless, it is worth noting that not all patients are capable of producing malignant pleural effusions, and additional studies are needed to explore the technique.

## Conclusions and future prospects

Organoids have emerged as one of the most promising biomedical technologies because of their high correlations with in vitro characteristics and drug response. Additional research into these models has resulted in emergence of organoid technology, which is now used for disease modelling, personalized medicine, drug development and screening. The high correlation between organoid-based drug response and clinical outcome has been revealed by real-world studies and clinical trials [92, 93]. Consequently, organoids have become a promising platform for the development of new anticancer drugs. Nevertheless, application of LCOs is beset by challenges, and the extent to which organoids can be assessed in vivo for predicting clinical response is yet to be fully investigated for different types of anti-cancer agent. Previous studies have primarily focused on investigating tumour cells and tissues, with limited research conducted on the biological behavior of patients, including cancer progression and treatment response. Consequently, evaluating the efficacy of organoid models in patients has emerged as a crucial concern in current research. Moreover, appraisal of immunotherapeutic drugs, such as immune checkpoint inhibitors, is still underway. At present, application of organoid techniques is advancing from the laboratory to the clinical setting as an option for individualized medicine approaches to find adequate treatments; nevertheless, there is still a considerable journey ahead.

To establish reliable organoid models, researchers should focus on combining organoids with advanced technologies and ensuring their validity in real-world environments. Forthcoming research on organoids in lung cancer will concentrate on various aspects. First, establishment of standards for organoid culture, quality control, and drug testing is still necessary. Automatic approaches for organoid culture and drug tests will be investigated, such as microfluidic chips and automated workstations, to reduce the instability due to manual operations. Moreover, adoption of a complex model that encompasses the vascular system, immune cells, fibroblasts, and other components to simulate the tumour microenvironment is indispensable for advancing the biological significance and accuracy of in vitro tests. As the culture techniques of LCOs progressively develop, this model will indubitably assume greater significance in personalized medicine, drug development and basic research of lung cancer.

## Data Availability

Not applicable.
